# Assessment of the Implementation of Critical Pathway in Stroke Patients: A 10-Year Follow-Up Study

**DOI:** 10.1155/2020/3265950

**Published:** 2020-02-26

**Authors:** Yun Jeong Jang, Dahye Park, Hyeong Seop Kim, Chang Han Lee, Ha Young Byun, Chul Ho Yoon, Eun Shin Lee, Heesuk Shin, Se-Woong Chun, Seung-Kyu Lim, Min-Kyun Oh

**Affiliations:** ^1^Department of Rehabilitation Medicine, Gyeongsang National University School of Medicine and Gyeongsang National University Hospital, Jinju 52727, Republic of Korea; ^2^Gyeongnam Regional Cardiocerebrovascular Disease Center, Jinju 52727, Republic of Korea; ^3^Department of Rehabilitation Medicine, Gyeongsang National University School of Medicine and Gyeongsang National University Changwon Hospital, Changwon 51472, Republic of Korea

## Abstract

**Background:**

The complications after stroke inhibit functional recovery and worsen the prognosis of patients. The implementation of a critical pathway (CP) can facilitate functional recovery after stroke by enabling comprehensive and systematic structured rehabilitation.

**Objective:**

To evaluate the effects of the implementation of CP in stroke patients for 10 years.

**Methods:**

The data were collected from 960 patients who were diagnosed with a stroke at the university hospital emergency room, who were transferred to the rehabilitation center after the acute phase, and who were discharged after undergoing comprehensive rehabilitation. Based on data collected over a period of 10 years, changes in demographic and stroke characteristics, preexisting medical conditions, poststroke complications, and functional states, as well as length of stay (LOS), were evaluated before and after CP implementation. The modified Rankin Scale (mRS) and the Korean version of the Modified Barthel Index (K-MBI) were used to evaluate functional states.

**Results:**

There were no significant differences in demographic and stroke characteristics before and after CP implementation. For those with preexisting medical conditions, there was no significant difference between before and after CP implementation. The majority of the complications were significantly decreased after the implementation of CP. Except for hemorrhagic stroke patients, the Brunnstrom stage in the ischemic and total stroke patients after CP implementation was significantly increased in the upper and lower extremities. The total hospitalization LOS and rehabilitation center hospitalization times were significantly reduced in ischemic and total stroke patients. There was no statistically significant difference in the functional gain of K-MBI and the efficiency of rehabilitation between before and after CP implementation.

**Conclusion:**

The implementation of CP allows for better application of evidence- and guideline-based key interventions and helps to provide early, comprehensive, organized, and more specialized care to stroke patients. Despite limited evidence, CP is still recommended as a means of promoting best practices in hospital care for stroke patients.

## 1. Introduction

Stroke has been a major cause of death and disability of people over 65 years old. In Korea, the fast migration toward aging society accompanies increasing cases of stroke and causes rising economic burden [1, 2]. The stroke accompanies most of complication onset within the first 6 weeks, and the complications inhibit functional recoveries of senile people thereby resulting in poor prognoses [3]. Thus, researches on finding ways, enabling systematic and comprehensive treatment including specialized medical teams and facilities, to reduce the occurrence of complications and to promote functional recovery of patients, have been in progress [3].

Medical teams strive to provide the objective and accurate medical services for stroke patients, and the structured comprehensive treatment plans, the critical pathways (CP), are implemented to give patients proper treatment sequentially [4, 5]. The implementation of CP enables patients to have treatments based on evidences as well as to secure efficiency of treatment to result in favorable clinical outcomes [6]. Besides, the implementation of CP grants a standardized protocol including medication, examination, procedure, and rehabilitation, together with a foundation for providing medical services integrated efficiently for patients in hospitals of the acute phase and rehabilitation and in long-term nursing facilities and home [6, 7].

The implementation of CP can raise the level of communication between medical teams and effect of rehabilitation, and it has been known that it can minimize treatment delay, reduce length of stay in the hospital and incidence of complications on patients, and decrease treatment cost [6–8]. In addition, it has been known that the patients who were taken care of through CP exhibited a higher level of reliability on treatment, together with an improvement in quality and results of treatment [8, 9].

In the studies that employed CP for the rehabilitation of patients suffering from stroke, Falconer et al. reported that they found no effect of the implementation of CP pertinent to stroke, whereas Sulch et al. reported an improvement in the functional state and quality of life from a randomized controlled trial, and Lim et al. reported the decreased length of stay in the hospital from their study based on the modified Rankin Scale (mRS) [10–12]. Kim et al. conducted the study on effect of implementation of CP in a university hospital; however, there were no studies on the long-term follow-up of the effect of implementation of CP [13].

The present study is intended to find long-term effect of implementation of CP for inpatients suffering from stroke in the department of rehabilitation medicine in a university hospital. The data accumulated for 10 years were used for comparative analysis on varied complications, demographic and stroke characteristics, preexisting medical conditions, functional states, site of discharge, and length of stay of patients before and after implementation of CP.

## 2. Materials and Methods

### 2.1. Subjects

The 960 patients diagnosed with ischemic or hemorrhagic stroke through examinations of brain computed tomography or magnetic resonance imaging, and based on findings obtained from patients' history and physical examination, and transferred and discharged from the department of rehabilitation medicine upon completion of treatment of the acute phase followed by comprehensive rehabilitation treatment, were selected as subjects of the present study among patients who came to the emergency room of OO university hospital. A total of 100 patients who came to the hospital during the period from January 1, 2008, to December 31, 2009, and then transferred to the department of rehabilitation medicine, were classified as patients of the group before implementation of CP, whereas the 860 patients who came to the hospital during the period from January 1, 2010, to February 28, 2019, and were transferred and discharged from the department of rehabilitation medicine were classified as patients of the group after implementation of CP.

**Figure 1:**
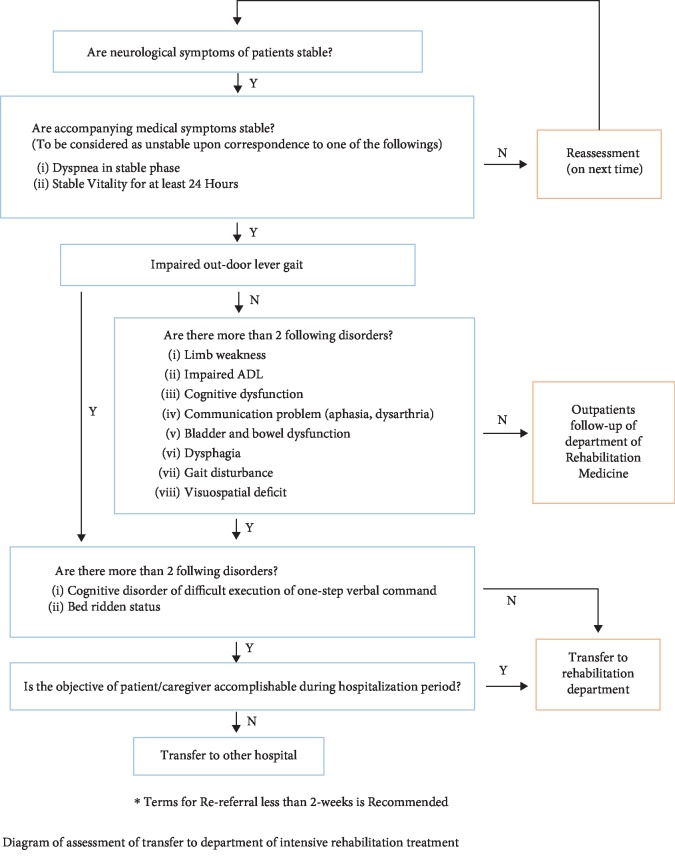
Critical pathway on comprehensive rehabilitation and rehabilitation department transfer in stroke patients. Impaired ADL: impaired activities of daily living; Y: yes; N: no.

For the treatment of patients in an acute phase of stroke through CP, the early rehabilitation program including prevention of complications was implemented upon assessment based on physical examination, degree of severity, functional state, and presence of complications through collaboration with the department of rehabilitation medicine within 3 days from the onset of stroke. In the early rehabilitation program, the patients, who manifested neurological stability, were supposed to transfer to the department of rehabilitation medicine for comprehensive treatment or discharged as outpatient upon the appraisal of transferability, and in cases of the progression of stroke in the course of treatment, the state of patients was reappraised for treatment pertinent to corresponding states of patients (Figure 1). The present study was approved by IRB of OO university hospital (IRB No. 2019-08-027).

### 2.2. Methods

Prior to CP implementation, a neurologist or neurosurgeon evaluated the patients' condition and referred them to the department of rehabilitation for rehabilitation treatment. After the implementation of CP, it is necessary that rehabilitation physicians assessed stroke patients early and conducted comprehensive rehabilitation through an evidence-based care protocol.

Medical records of subjects were examined retrospectively. Features of stroke and demographics before and after implementation of CP including age, sex, types of causes of stroke (ischemic or hemorrhagic), sides of hemiplegia (right, left, or both) due to stroke, and location of lesion (cortical lesion, subcortical lesion, and infratentorial lesion) were examined. Underlying medical diseases of patients such as hypertension, abuse of alcohol, diabetes mellitus, smoking, cardiac arrhythmia, osteoarthritis, dyslipidemia, previous stroke, coronary artery disease, valvular heart disease, renal failure, chronic obstructive pulmonary disease or asthma, deep vein thrombosis, hydrocephalus, and history of seizure were examined.

Following complications of stroke that occurred before discharge from the department of rehabilitation medicine were also examined: poststroke shoulder pain, dysphagia, poststroke depression, Foley catheter insertion, pneumonia, urinary tract infection, hydrocephalus, adverse effects of drugs, tracheostomy, progression of stoke, gastrointestinal symptoms (gastritis, gastric ulcer, gastric hemorrhage, and enteritis), electrolyte imbalance, deep vein thrombosis, fall, poststroke seizure, nutrition supply through gastrostomy (percutaneous endoscopic gastrostomy), decubitus ulcer, and heterotopic ossification.

All subjects were classified into patients of ischemic and hemorrhagic strokes to appraise the functional state of each patient, and the mRS and Brunnstrom stage on proximal and distal upper limbs and lower limbs, before and after transfer to the department of rehabilitation medicine, were examined. And the Korean version of the Modified Barthel Index (K-MBI) was examined upon transfer to and discharge from the department of rehabilitation medicine to calculate the functional gain (scores at discharge–scores on transfer to department of rehabilitation medicine) and efficiency of rehabilitation (functional gain/length of stay in department of rehabilitation medicine).

To identify the effect of implementation of CP upon length of stay in the hospital of patients, the periods from the onset of stroke to initiation of early rehabilitation treatment, of length of stay before transfer to the department of rehabilitation medicine, of length of stay in the department of rehabilitation medicine, and of overall length of stay in the hospital were examined.

Besides, the transfer of patients to home or other hospitals upon discharge from the department of rehabilitation medicine was also examined to appraise the return to society after rehabilitation. Other hospitals mentioned here include the short-term general hospital with rehabilitation department, specialized rehabilitation hospital, and nursing homes.

### 2.3. Statistical Analysis

An independent *t*-test was performed to compare the mean values of variables between two independent groups (pre-CP and post-CP). Variables are mRS, Brunnstrom stage, K-MBI, and length of stay in the hospital. Levene's test was performed for homogeneity of the variance test. In the independent *t*-test, when the significance level was 5%, the significance probability values of all variables were greater than the significance level. The basic assumptions (normal distribution, homogeneity of variance) for the population were satisfied, so the parametric statistic methods were used.

The data on sex, cause of stroke, location of lesion, sides of hemiplegia due to stroke, past medical history of patients, complications occurring before discharge from the hospital, and types of discharge were comparatively analyzed by conducting a Chi-squared test. SPSS version 21.0 for Windows (IBM, Armonk, NY, USA) was employed for all statistical analyses conducted in the present study with the level of statistical significance of *p* < 0.05.

## 3. Results

### 3.1. Demographic and Stroke Characteristics

Among all 960 subjects, the subjects belonging to the group before implementation of CP were 100 subjects wherein 53 male subjects (53.0%) and 47 female subjects (47.0%) were included; the average age of the 100 subjects was 62.0 ± 13.1 years. 860 subjects belonging to the group after implementation of CP comprised 433 male subjects (50.3%) and 427 female subjects (49.7%), and the average age of the group was 63.9 ± 12.9 years.

Regarding the types of stroke of subjects, the stroke ascribable to ischemic causes comprised 48 subjects (48.0%) and the stroke ascribable to hemorrhagic causes comprised 52 subjects (52.0%) before implementation of CP. The stroke attributable to ischemic causes comprised 412 subjects (47.9%), and the stroke attributable to hemorrhagic causes comprised 448 subjects (52.1%) after implementation of CP. Sides of hemiplegia before implementation of CP consisted of 49 subjects of right side (49.0%), 36 subjects of left side (36.0%), and 15 subjects of both sides (15.0%), whereas 349 subjects of right side (40.6%), 379 subjects of left side (44.1%), and 132 subjects of both side (15.3%) were in the group after implementation of CP. Locations of lesions of stroke comprised 3 subjects of cortical lesion (3.0%), 10 subjects of infratentorial lesion (10.0%), and 87 subjects of subcortical lesion (87.0%) before implementation of CP, whereas 37 subjects of cortical lesion (4.3%), 140 subjects of infratentorial lesion (16.3%), and 683 subjects of subcortical lesion (79.4%) were in the group of subjects after implementation of CP. The proportion of the cortex lesion was very lower than that of the subcortical lesion. This is because cortical engagement was included only when the lesions were confined to the cortex. Subcortical engagement included both cortical and subcortical lesions as well as those limited to subcortical. There were no significant differences between the two groups (Table 1).

### 3.2. Preexisting Medical Conditions

The underlying medical diseases of subjects before implementation of CP appeared in the following order of 70 subjects of hypertension (70.0%), 38 subjects of alcohol abuse (38.0%), 33 subjects of diabetes mellitus (33.0%), 31 subjects of smoking (31.0%), 28 subjects of cardiac arrhythmia (28.0%), 25 subjects of osteoarthritis (25.0%), 25 subjects of dyslipidemia (25.0%), and 20 subjects of previous stroke (20.0%), whereas that of subjects after implementation of CP appeared in the following order: 640 subjects of hypertension (74.4%), 365 subjects of alcohol abuse (42.4%), 236 subjects of diabetes mellitus (27.4%), 221 subjects of smoking (25.7%), 221 subjects of osteoarthritis (25.7%), 144 subjects of cardiac arrhythmia (16.7%), 145 subjects of past history of stroke (16.9%), and 104 subjects of dyslipidemia (12.1%). The two groups exhibited significant decreases of cardiac arrhythmia from 28.0% to 16.7% and dyslipidemia from 25.0% to 12.1%; no significant differences in the distribution of other underlying medical diseases between two groups were found (Table 2).

### 3.3. Complications

78 subjects of poststroke shoulder pain (78.0%) and 66 subjects of poststroke dysphagia (66.0%) appeared over half of the total population of the group of subjects before implementation of CP; these were followed by the appearance of the following complications: 46 subjects of depression (46.0%), 38 subjects of Foley catheter insertion (38.0%), 32 subjects of pneumonia (32.0%), and 23 subjects of urinary tract infection (23.0%). Upon implementation of CP, poststroke complications appeared in the following order: 461 subjects of dysphagia (53.6%), 389 subjects of shoulder pain (45.2%), 192 subjects of Foley catheter insertion (22.3%), 169 subjects of depression (19.7%), and 122 subjects of gastrointestinal symptoms (14.2%). Except for gastrointestinal symptoms, nutrition supply through gastrostomy (percutaneous endoscopic gastrostomy), decubitus ulcer, and heterotopic ossification, the appearance of poststroke complications decreased significantly with the implementation of CP (Table 3).

### 3.4. Functional Evaluation

In the group of subjects of total stroke, the Brunnstrom stages in proximal and distal upper extremity and lower extremity were 3.4 ± 1.2, 3.4 ± 1.3, and 3.4 ± 1.1, respectively, before implementation of CP. The stages were significantly increased to 3.9 ± 1.2, 3.8 ± 1.3, and 4.0 ± 1.1, respectively, along with the implementation of CP. In the group of subjects of ischemic stroke, the Brunnstrom stages in proximal and distal upper extremity and lower extremity also manifested statistically significant increase upon implementation of CP; however, the increase was not statistically significant for the hemorrhagic stroke.

The group of subjects of total stroke and the groups distinguished by ischemic and hemorrhagic strokes did not show statistically significant differences of functional gain and efficiency of rehabilitation reflected in terms of mRS, K-MBI at transfer, and K-MBI at discharge (Table 4).

### 3.5. Length of Stay

Upon implementation of CP, the times of the period from the onset of stroke to initiate rehabilitation treatment and of the length of stay from the onset of stroke to transfer to the department of rehabilitation medicine decreased significantly for the groups of subjects of total, ischemic, and hemorrhagic strokes.

The length of stay in the department of rehabilitation medicine and the total length of stay in the hospital for the groups of subjects of total and ischemic strokes commonly exhibited significant decreases; however, the group of subjects of hemorrhagic stroke did not show statistically significant differences in length of stay (Table 5).

### 3.6. Site of Discharge

The site of discharge of subjects was distinguished into discharge to home and discharge to transfer to other hospitals. The group of subjects of ischemic stroke showed 6 cases of discharge to home (12.5%) and 42 cases of discharge to other hospitals (87.5%) before implementation of CP, whereas the site of discharge to home increased significantly with the following figures: 175 cases of discharge to home (42.5%) and 237 cases of discharge to other hospitals (57.5%), upon implementation of CP. The groups of subjects of total and hemorrhagic strokes did not exhibit statistically significant differences in the site of discharge (Table 6).

## 4. Discussion

The present study is intended to identify long-term effect of implementation of CP for rehabilitation prepared for inpatients of stroke in the university hospital. Although there are many previous studies on ischemic stroke, few studies have analyzed hemorrhagic stroke despite the high proportion of hemorrhagic stroke accounting for total stroke [14]. Therefore, data were classified into ischemic and hemorrhagic strokes and analyzed.

The medically underlying diseases such as hypertension, abuse of alcohol, diabetes mellitus, and smoking appeared high before and after implementation of CP. These were similar to results of previous study that presented hypertension, smoking, diabetes mellitus, and dyslipidemia as risk factors of stroke [1]. The cardiac arrhythmias and dyslipidemia of subjects of stroke appeared significantly decreasing upon implementation of CP compared to before implementation of CP; regarding the report that pointed out the significant difference in frequency of diagnosis depending on environment of treatment of stroke [15], and the characteristic of present study employing a retrospective approach to medical records, the difference can be attributable to the resulting degree of integrity of medical records.

In the present study, the significant decrease in the rate of occurrence of most of the complications in the group of subjects suffering from stroke was found upon implementation of CP, wherein the complications of shoulder pain and dysphagia were found dominantly from both groups. The significant decrease in the rate of occurrence of aspiration pneumonia seems attributable to the early assessment and treatment for dysphagia upon implementation of CP. The results agree with those of previous studies that reported the decreases in the rate of onset of pneumonia, length of stay, and mortality by early diagnosis and treatment of dysphagia [16]. The onset of stroke may accompany shoulder pain on paralytic side due to reduced muscle strength or contracture of soft tissue. Early rehabilitation treatment was initiated upon implementation of CP, and the education on appropriate postures in bed, wearing of an arm sling in cases of severe paralysis, exercises of articular motion of the shoulder joint, and exercises of muscle force seem ascribable to the decrease and prevention of poststroke shoulder pain. These agree with those findings of higher functional independence and decrease in the onset of complications by an early rehabilitation compared to ordinary rehabilitation which were reported in results of prior studies [17, 18].

Poststroke depression also appeared significantly decreased in the group of patients after implementation of CP. Appropriate management through psychotherapy and drug medication upon diagnosis of patients' symptoms are recommended for cases of poststroke depression that could decrease functional rehabilitation and participation in rehabilitation treatment of patients; the early mobilization has been known to alleviate the depressive mood of patients of stroke [19, 20]. Upon implementation of CP, the patients were provided with education, psychotherapy, and drug treatment from early diagnoses made through screening at an early stage of stroke together with early mobilization, which were estimated to be attributable to the decrease in the onset of poststroke depression.

A Foley catheter is used in cases of urinary dysfunction due to brain damages from stroke or for operation and examination thereof, and it has been known that complications such as urinary tract infection due to the insertion of a Foley catheter together with urinary incontinence and neurogenic bladder dysfunction occur frequently [3, 21]. The use of a Foley catheter and complications including urinary tract infection decreased significantly upon implementation of CP compared to those before implementation of CP. The consequence seems attributable to the implementation of a bladder training program in cases of urinary dysfunction during the period of comprehensive rehabilitation and clean intermittent catheterization or removal of the Foley catheter to reduce risk of urinary tract infection through assessment of urinary function in the early stage of rehabilitation treatment.

Fall and deep vein thrombosis were decreased significantly due to the increase in early mobilization and education on fall and postures in bed in the early rehabilitation besides the complications mentioned earlier. And the occurrence of complications of hydrocephalus, poststroke seizure, and electrolyte imbalance together with progression of stroke was decreased by identifying the presence of problems through examinations on blood and brain images and by the use of anticonvulsant pertinent to the reduction of risk of seizure upon early assessment of patients. These consequences correspond to findings of reduced occurrence of poststroke complications through an intervention of early treatment pertinent to rehabilitation [8, 21, 22].

The mRS score tended to decrease in the groups of subjects of total, ischemic, and hemorrhagic strokes upon implementation of CP with the transfer to the department of rehabilitation medicine, whereas the Brunnstrom stage increased significantly in the groups of subjects of total and ischemic strokes. The results correspond to the result of previous study that reported that a comprehensive assessment and implementation of early rehabilitation plan would help functional rehabilitation and development of discharge plan of patients [23] as well as early rehabilitation for patients suffering from stroke that intended for higher functional recovery [13, 17].

On the contrary, the group of subjects of hemorrhagic stroke exhibited no statistically significant differences in terms of the Brunnstrom stage. This was estimated that it would be attributable to difficulties in making early active treatment of rehabilitation due to postoperative treatment in the intensive care unit (ICU) and delay in initiation of comprehensive rehabilitation for the group of patients of hemorrhagic stroke who are frequently in need of a surgical procedure.

The score of K-MBI upon transfer to and discharge from the department of rehabilitation medicine tended to be increasing upon implementation of CP in the groups of subjects of total and ischemic strokes though it was not statistically significant, and the scores of functional gain and efficiency of rehabilitation also tended to be increasing though they were not statistically significant. The group of subjects of hemorrhagic stroke exhibited a decreasing score of K-MBI upon transfer to and discharge from the department of rehabilitation medicine though it was not statistically significant; this was estimated that it would be ascribable to a higher rate of occurrence of poststroke complications in the group of subjects of hemorrhagic stroke. The consequence corresponds to the result of study that reported increasing risk of onset of complications by the sustainment of invasive procedure in ICU of most of the patients suffering from hemorrhagic stroke [24]. Functional gain tended to be increasing in the group of subjects of hemorrhagic stroke; however, the efficiency of rehabilitation tended to be decreasing though it was not statistically significant; this was estimated that it would be attributable to longer length of stay in the department of rehabilitation medicine compared to that of the group of ischemic stroke.

From the onset of stroke, the times to points of initiation of rehabilitation and of transfer to the department of rehabilitation medicine upon implementation of CP appeared significantly shortened compared to those before implementation of CP in the groups of subjects of total, ischemic, and hemorrhagic strokes. This was estimated to be a consequence that resulted from early assessment and rehabilitation upon implementation of CP. The length of stay in the hospital also manifested a significant decrease in groups of subjects of total and ischemic strokes upon implementation of CP; this was attributed to the decrease in the rate of occurrence of poststroke complications. In the previous study, the complications that occurred on inpatients during rehabilitation have been known as a strong prognostic factor of increasing length of stay [25]. On the contrary, most of the group of hemorrhagic stroke patients took operative treatment compared to patients suffering from ischemic stroke and exhibited no significant decrease in the length of stay in the hospital due to admission into ICU for postoperative care and treatment for additional complications [24].

The cases of discharge to home upon implementation of CP increased significantly in the group of subjects of ischemic stroke compared to those of before implementation of CP. This was estimated that it would be ascribable to the decrease in incidence of complications and increase in the Brunnstrom stage, which is increased functional recovery.

The limitations of this study included the following: first, the retrospective approach that relied on medical records might have influenced the result of the present study depending on integrity of medical records. Second, the deficiency in items of comparative analysis due to differences in systems of medical records before and after implementation of CP resulted in the limited 100 samples for the control group before implementation of CP which are smaller than 860 instances after implementation of CP. Thirdly, there were differences between hospitals regarding points of initiation of rehabilitation and transfer to the department of rehabilitation medicine and length of stay, and these might have resulted in different consequences in hospitals that implemented the CP. Thus, the development of a standardized critical pathway based on large-scaled multi-institutional research seems necessary.

The academic significance of the present study resides in the long-term tracking and follow-up for more than 10 years of effect of implementation of CP upon poststroke complications, functional states, and length of stay of patients in the department of rehabilitation medicine for the first time. The results of the present study are expected to be exploited for the clinical application and as a foundation of implementation of CP intending for rehabilitation of patients suffering from stroke.

## 5. Conclusions

Implementation of a critical pathway promotes the key intervention based on evidences and guidelines, as well as helps provide patients with comprehensive, systematic, and professional treatment in the early times. The implementation of a critical pathway is recommended as a means of promoting the best treatment for patients suffering from stroke despite limited evidences.

## Figures and Tables

**Table 1:** Comparison of demographic and stroke characteristics before and after CP implementation.

	Before (*N* = 100)	After (*N* = 860)	*p* value
Age	62.0 ± 13.1	63.9 ± 12.9	0.163
Gender			0.692
Male	53 (53.0%)	433 (50.3%)	
Female	47 (47.0%)	427 (49.7%)	
Cause of stroke			1.000
Hemorrhage	52 (52.0%)	448 (52.1%)	
Infarction	48 (48.0%)	412 (47.9%)	
Side of hemiplegia			0.236
Right	49 (49.0%)	349 (40.6%)	
Left	36 (36.0%)	379 (44.1%)	
Bilateral	15 (15.0%)	132 (15.3%)	
Location of lesion			0.195
Cortical	3 (3.0%)	37 (4.3%)	
Infratentorial	10 (10.0%)	140 (16.3%)	
Subcortical	87 (87.0%)	683 (79.4%)	

Values are presented as the mean ± standard deviation or number (%). CP: critical pathway. ^∗^*p* < 0.05, ^∗∗^*p* < 0.01, and ^∗∗∗^*p* < 0.001.

**Table 2:** Comparison of preexisting medical conditions of stroke patients before and after CP implementation.

	Before (*N* = 100)	After (*N* = 860)	*p* value
Hypertension	70 (70.0%)	640 (74.4%)	0.405
Alcohol abuse	38 (38.0%)	365 (42.4%)	0.456
Diabetes mellitus	33 (33.0%)	236 (27.4%)	0.292
Smoking	31 (31.0%)	221 (25.7%)	0.307
Cardiac arrhythmia	28 (28.0%)	144 (16.7%)	0.008^∗∗^
Osteoarthritis	25 (25.0%)	221 (25.7%)	0.976
Dyslipidemia	25 (25.0%)	104 (12.1%)	0.001^∗∗^
Previous stroke	20 (20.0%)	145 (16.9%)	0.517
Coronary artery disease	12 (12.0%)	76 (8.8%)	0.393
Valvular heart disease	6 (6.0%)	76 (8.8%)	0.440
Renal failure	5 (5.0%)	34 (4.0%)	0.815
COPD/asthma	4 (4.0%)	34 (4.0%)	1.000
Deep vein thrombosis	2 (2.0%)	41 (4.8%)	0.312
Hydrocephalus	2 (2.0%)	9 (1.0%)	0.725
Seizure history	0 (0.0%)	6 (0.7%)	0.867

Values are presented as the number (%). CP: critical pathway; COPD: chronic obstructive pulmonary disease. ^∗^*p* < 0.05, ^∗∗^*p* < 0.01, and ^∗∗∗^*p* < 0.001.

**Table 3:** Comparison of the number of stroke patients with complications before and after CP implementation.

	Before (*N* = 100)	After (*N* = 860)	*p* value
Poststroke shoulder pain	78 (78.0%)	389 (45.2%)	0.000^∗∗∗^
Dysphagia	66 (66.0%)	461 (53.6%)	0.024^∗^
Poststroke depression	46 (46.0%)	169 (19.7%)	0.000^∗∗∗^
Foley catheter insertion	38 (38.0%)	192 (22.3%)	0.001^∗∗^
Pneumonia	32 (32.0%)	82 (9.5%)	0.000^∗∗∗^
Urinary tract infection	23 (23.0%)	80 (9.3%)	0.000^∗∗∗^
Hydrocephalus	21 (21.0%)	51 (5.9%)	0.000^∗∗∗^
Drug reaction	20 (20.0%)	74 (8.6%)	0.001^∗∗^
Tracheostomy	20 (20.0%)	77 (9.0%)	0.001^∗∗^
Stroke progression	19 (19.0%)	33 (3.8%)	0.000^∗∗∗^
GI symptoms	17 (17.0%)	122 (14.2%)	0.544
Electrolyte abnormality	12 (12.0%)	17 (2.0%)	0.000^∗∗∗^
Deep vein thrombosis	8 (8.0%)	27 (3.1%)	0.030^∗^
Fall	8 (8.0%)	12 (1.4%)	0.000^∗∗∗^
Poststroke seizure	7 (7.0%)	13 (1.5%)	0.001^∗∗^
PEG	5 (5.0%)	16 (1.9%)	0.095
Decubitus ulcer	3 (3.0%)	34 (4.0%)	0.846
Heterotopic ossification	2 (2.0%)	3 (0.3%)	0.151

Values are presented as the number (%). CP: critical pathway; GI: gastrointestinal; PEG: percutaneous endoscopic gastrostomy. ^∗^*p* < 0.05, ^∗∗^*p* < 0.01, and ^∗∗∗^*p* < 0.001.

**Table 4:** Comparison of functional states at transfer to rehabilitation center and discharge before and after CP implementation.

	Before	After	*p* value
Total	*N* = 100	*N* = 860	
mRS	4.1 ± 1.0	4.0 ± 1.0	0.237
Br-stage UE proximal	3.4 ± 1.2	3.9 ± 1.2	0.000^∗∗∗^
Br-stage UE distal	3.4 ± 1.3	3.8 ± 1.3	0.005^∗∗^
Br-stage LE	3.4 ± 1.1	4.0 ± 1.1	0.000^∗∗∗^
K-MBI (at transfer)	35.8 ± 23.3	36.2 ± 23.1	0.863
K-MBI (at discharge)	55.9 ± 28.8	57.6 ± 27.1	0.567
K-MBI gain^a^	20.2 ± 16.3	21.4 ± 17.7	0.510
K-MBI efficiency^b^	0.6 ± 0.7	0.6 ± 0.7	0.475
Infarction	*N* = 48	*N* = 412	
mRS	3.7 ± 1.0	3.6 ± 1.1	0.344
Br-stage UE proximal	3.2 ± 1.2	4.1 ± 1.1	0.000^∗∗∗^
Br-stage UE distal	3.2 ± 1.3	3.9 ± 1.2	0.000^∗∗∗^
Br-stage LE	3.2 ± 1.1	4.3 ± 1.0	0.000^∗∗∗^
K-MBI (at transfer)	35.9 ± 23.0	40.8 ± 22.0	0.143
K-MBI (at discharge)	54.8 ± 28.3	61.0 ± 25.4	0.116
K-MBI gain^a^	18.9 ± 15.1	20.1 ± 16.2	0.615
K-MBI efficiency^b^	0.6 ± 0.9	0.8 ± 0.9	0.132
Hemorrhage	*N* = 52	*N* = 448	
mRS	4.5 ± 0.7	4.4 ± 0.7	0.342
Br-stage UE proximal	3.5 ± 1.2	3.6 ± 1.3	0.467
Br-stage UE distal	3.5 ± 1.3	3.6 ± 1.3	0.769
Br-stage LE	3.6 ± 1.1	3.8 ± 1.2	0.270
K-MBI (at transfer)	35.7 ± 23.7	31.9 ± 23.2	0.142
K-MBI (at discharge)	57.0 ± 29.4	54.5 ± 28.2	0.066
K-MBI gain^a^	21.3 ± 17.4	22.6 ± 19.0	0.661
K-MBI efficiency^b^	0.6 ± 0.6	0.5 ± 0.5	0.320

Values are presented as the mean ± standard deviation. CP: critical pathway; mRS: modified Rankin Scale; Br-stage: Brunnstrom stage; UE: upper extremity; LE: lower extremity; K-MBI: Korean version of the Modified Barthel Index. ^a^Function at discharge: function at rehabilitation start. ^b^Gain/rehabilitation stays (days). ^∗^*p* < 0.05, ^∗∗^*p* < 0.01, and ^∗∗∗^*p* < 0.001.

**Table 5:** Comparison of length of stay (LOS) before and after CP implementation.

	Before	After	*p* value
Total	*N* = 100	*N* = 860	
Time from stroke onset to RT start	19.9 ± 20.0	5.5 ± 8.9	0.000^∗∗∗^
Time from onset to RH transfer day	42.4 ± 36.0	22.3 ± 15.5	0.000^∗∗∗^
RH LOS	64.5 ± 62.6	45.9 ± 32.9	0.004^∗∗^
Total LOS	84.3 ± 58.6	68.2 ± 42.7	0.009^∗∗^
Infarction	*N* = 48	*N* = 412	
Time from stroke onset to RT start	11.2 ± 12.1	1.5 ± 0.9	0.000^∗∗∗^
Time from onset to RH transfer day	31.0 ± 22.7	15.7 ± 10.2	0.000^∗∗∗^
RH LOS	57.2 ± 43.4	32.3 ± 25.4	0.000^∗∗∗^
Total LOS	85.9 ± 54.8	47.9 ± 30.5	0.000^∗∗∗^
Hemorrhage	*N* = 52	*N* = 448	
Time from stroke onset to RT start	27.8 ± 22.5	9.2 ± 11.2	0.000^∗∗∗^
Time from onset to RH transfer day	53.0 ± 42.5	28.4 ± 17.0	0.000^∗∗∗^
RH LOS	71.2 ± 76.0	58.5 ± 34.1	0.236
Total LOS	82.8 ± 62.5	86.8 ± 43.9	0.652

Values are presented as the mean ± standard deviation (days). CP: critical pathway; RT: rehabilitation therapy; RH: rehabilitation; LOS: length of stay. ^∗^*p* < 0.05, ^∗∗^*p* < 0.01, and ^∗∗∗^*p* < 0.001.

**Table 6:** Comparison of site of discharge before and after CP implementation.

	Before	After	*p* value
Total	*N* = 100	*N* = 860	
Site of discharge			0.081
Home	23 (23.0%)	276 (32.1%)	
Other hospitals	77 (77.0%)	584 (67.9%)	
Infarction	*N* = 48	*N* = 412	
Site of discharge			0.000^∗∗∗^
Home	6 (12.5%)	175 (42.5%)	
Other hospitals	42 (87.5%)	237 (57.5%)	
Hemorrhage	*N* = 52	*N* = 448	
Site of discharge			0.145
Home	17 (32.7%)	101 (22.5%)	
Other hospitals	35 (67.3%)	347 (77.5%)	

Values are presented as the number (%). CP: critical pathway. ^∗^*p* < 0.05, ^∗∗^*p* < 0.01, and ^∗∗∗^*p* < 0.001.

## Data Availability

The data used to support the findings of this study are available from the corresponding author upon request.
